# The Woman Worker's World

**Published:** 1918-10-19

**Authors:** 


### THE WOMAN WORKER'S WORLD.

Talks to Mothers.

*Maternity and Child Welfare* * keeps up its high standard, and for a monthly sixpence gives excellent articles on practical work for the children's good. The September number contains a reprint of Dr. Raimondi's interesting paper on four years' work in France, read during the Baby Conference. Two very helpful papers for social workers are Dr. Esther Carling's "Twenty Minutes' Talk on Tuberculosis," one of a series of such talks, and the account of a Prepared Discussion held in a school for mothers, given by Miss H. Cashmore, Warden of the University Settlement at Bristol. The plan of sending a paper of questions to each mother to be thought over for a week, and if possible discussed with her own friends before a meeting of members, is excellent. A cup of tea was handed round and the discussion briefly opened by the Chair. Each mother had her name written against one question on the paper sent her, and opinions came readily. The Health Visitor's account of a typical day in her life is also one of a series. It is curious to note how in all this literature, not least in the article by Dr. Constance Long on "The Psychic Life of the Child under Five," the same point emerges, the desperate need of attention to the housing problem as our very first post-war duty.

Homes for Working Girls in London.

Hostels are increasingly needed now, and it seems likely that they will not be less necessary after the war. There are few better ways of fighting the evils of our streets than that of providing girls earning their own living with fit dwelling-places where good influences surround them and a home feeling can exist, but yet there is no sense of "charity." At Mr. John Shrimpton's homes, now seven in number, since Morley House is to be closed, a working girl who is under twenty-five can be received and have bedroom and three meals a day, with use of sitting-room, library, and piano, for 6s. 6d. per week. The latest report contains some pathetic little histories which prove the boon a very great one, and contains also some charming details of happy life, such as the tea-party followed by a little "home-made" concert given to fifty old women by the girls in one home. We are sorry to see that Mr. Shrimpton comments on the intrusion of the fashion—we are tempted to call it—of pilfering and stealing from each other by young women. From Government offices, from Art Schools, from every kind of office and workshop we hear the same tale. In the present fashionable phrase "What about it?" The custom is not a pretty one. We are inclined to attribute it in part to the neglect of the Church Catechism, in part to the prevalent idea that each girl must dress better than all the rest, but perhaps still more to the habit of calling theft by other names. If one *pinches* a collar, *sneaks* a tube of paint, *annexes* a blouse, it is somehow quite different—perhaps!

The hostels ask for new subscribers (naturally they are not quite self-supporting) and books for the library. Mr. Shrimpton's address is 3 Victoria Street, S.W.

* *Maternity and Child Welfare.* 6d. monthly. (John Bale, 83 Great Titchfield Street, W. 1.)

